# Synthesis, Characterization and Bioactivity Evaluation of a Novel Nano Bagasse Xylan/Andrographolide Grafted and Esterified Derivative

**DOI:** 10.3390/polym14163432

**Published:** 2022-08-22

**Authors:** Kexin Tian, Heping Li, Bin Zhao, Yue Su, Zhiming Zou, Wenli Wang

**Affiliations:** 1 College of Chemistry and Bioengineering, Guilin University of Technology, Guilin 541004, China; 2 China National Textile and Apparel Council Key Laboratory of Natural Dyes, College of Textile and Clothing Engineering, Soochow University, Suzhou 215123, China; 3 National Engineering Laboratory for Modern Silk, College of Textile and Clothing Engineering, Soochow University, Suzhou 215123, China

**Keywords:** bagasse xylan/andrographolide derivative, synthesis, molecular docking, synergistic anticancer, composite nanomaterial

## Abstract

In the in-depth research that has been conducted on nanometer biomaterials, how to use the biomass resources with high activity and low toxicity to prepare nanomaterials for biomedical applications has attracted much attention. To realize efficient and comprehensive utilization of biomass, bagasse xylan/andrographolide (BX/AD) was ued as a raw material and glycyrrhetinic acid (GA) as an esterification agent to synthesize bagasse xylan/andrographolide esterified derivative (GA-BX/AD). Then, the bagasse xylan/andrographolide grafted and esterified derivative (GA-BX/AD-g-IA) was synthesized by the graft crosslinking reactions using itaconic acid (IA) as graft monomer. The better synthesis conditions were optimized by single factor experiments, the degree of esterification substitution (*DS*) was 0.43, and the grafting rate (*G*) of the product reached 42%. The structure and properties of the product were characterized by FTIR, XRD, DTG, SEM, and ^1^H NMR. The results showed that the product morphology was significantly changed, and the nanoparticles were spherical with a particle size of about 100 nm. The anti-cancer activity of the product was measured. The molecular docking simulations revealed that the product had good docking activity with human glucocorticoid protein (6CFN) with a binding free energy of 14.38 kcal/mol. The MTT assay showed that the product had a strong inhibitory effect on the growth of human liver cancer cells (BEL-7407) and gastric cancer cells (MGC80-3), with inhibition ratio of 38.41 ± 5.32% and 32.69 ± 4.87%. Therefore, this nanomaterial is expected to be applied to the development and utilization of drug carriers and functional materials.

## 1. Introduction

Cancer is one of the most terrible and serious health problems in the world [1,2]. Chemotherapy is the main method used to treat cancer, but long-term use of single-drug chemotherapy can easily make cancer cells resistant to chemotherapeutic drugs and produces large toxic and side effects [3]. Research shows that effective cancer treatment requires the combined use of multiple anticancer drugs, and reducing the dosage of each drug can achieve the same anticancer effect [4]. Therefore, exploring and developing natural products with small toxic and side effects as raw materials and modern structural modification will be expected to provide a new direction for the development of cancer drugs.

Plant polysaccharides have many biological functions, such as inhibiting the proliferation of tumor cells [5], improving the antioxidant capacity of substances [6], and enhancing biological immunity, et al. [7]. Among them, xylan is one of the polysaccharides that has unique biological activity and physiological functions [8], such as high solubility and good biocompatibility. The structure of xylan contains a large number of hydrophilic hydroxyl groups, which can be chemically modified by etherification [9,10], grafting [11], and crosslinking [12]. Studies have shown that xylan and its derivatives have certain immunomodulatory functions and anticancer activity [13,14], which can restore the immune system disorder caused by tumor cells and promote cell apoptosis. It is an effective supplement to traditional drug therapy. Choi et al. [15] sulfated rhamnose polysaccharide (SPS-CF) and tested its anticancer activity in vitro. The results showed that it could inhibit the spread of colon cancer by inducing apoptosis of cancer cells. Therefore, our study hopes to explore a method that can not only maintain the physical and chemical properties of xylan, but also improve its anticancer activity.

Andrographolide is the main active substance of Andrographis paniculate, which can inhibit the proliferation and induce apoptosis of cancer cells [16]. Swati et al. [17] found that it has cytotoxicity to almost all types of cancer cells. At the same time, andrographolide can also be modified by introducing different groups to reduce hydrophobicity and improve bioavailability. A series of thioether andrographolide derivatives were synthesized by etherification modification of andrographolide. The in vitro anticancer activity test showed that these derivatives had an inhibitory effect on breast cancer cells [18]. Liu et al. [19] synthesized a series of novel andrographolide sulfide derivatives by introducing aromatic substituents on C—12 and tested their anticancer activity in vitro. The experimental results showed that this series of derivatives had higher anticancer activity than andrographolide. 

In recent years, domestic and international scholars have realized the high-value application of biomass resources by preparing multifunctional composites [20,21]. A new pH-responsive prodrug nanoparticle was synthesized by combining xylan and curcumin. The result of the activity test shows that it has obvious cytotoxicity to human colon cancer cells HT-29 and HCT-15; that is, it has an obvious inhibitory effect on the proliferation of colon cancer cells [22]. At the same time, the synergistic advantages of nanocarrier and polymerization coupling were also proved. Yuan et al. [23] discussed the inhibitory effect of paclitaxel combined with andrographolide on lung cancer cells A549. The results showed that the inhibition rate of paclitaxel combined with andrographolide on cancer cells was 98%, which confirmed that andrographolide and paclitaxel had a synergistic anticancer effect. However, there have been no studies of andrographolide complexed with xylan against cancer. Studies have shown that xylan mainly plays an immunomodulatory role by improving specific and nonspecific immunity, inhibiting the proliferation and differentiation of cancer cells [24,25,26]. Andrographolide usually inhibits cell replication and migration by inhibiting the transmission of cancer cell growth signals and the production of transcription activators. Both might exert anticancer effects through different mechanisms, and if they were used in combination for the study of anticancer activity, synergistic effects would be expected.

Therefore, based on the above discussion, bagasse xylan was compounded with andrographolide for synergistic effect. A new bagasse xylan/andrographolide complex derivative was synthesized by using glycyrrhetinic acid as the esterifying agent and itaconic acid as the grafting monomer and introducing the reactive groups through esterification, grafting, and cross-linking based on the principle of free radical polymerization. The derivative was prepared as nanoparticles by nanoprecipitation and its anticancer activity was evaluated based on the biological properties of xylan and andrographolide.

## 2. Materials and Methods

### 2.1. Materials

BX was isolated from sugarcane bagasse (self-extracted). Andrographolide (AD) and glycyrrhetinic acid (GA) were obtained from Jiuding Chemical Company (Shanghai, China). Thionyl chloride (SOCl_2_) was purchased from Luoyang Chemical Reagent Factory (Luoyang, China). Ammonium persulphate (APS) was purchased from Chemre Chemical Reagent Development Centre (Tianjin, China). N, N-methylene-bisacrylamide (MBAA) was produced by Xilong Chemical Company (Shantou, China). Absolute ethanol and N, N-dimethylformamide (DMF) were purchased from Kaitong Chemical Reagent Factory (Tianjin, China). Dimethyl sulfoxide (DMSO) originated from Paini Chemical Reagent Factory (Zhengzhou, China). 

### 2.2. Synthesis of Bagasse Xylan/Andrographolide Grafted and Esterified Derivative 

#### 2.2.1. Synthesis of Glycyrrhetinic Acid Chloride

**Figure 1:**
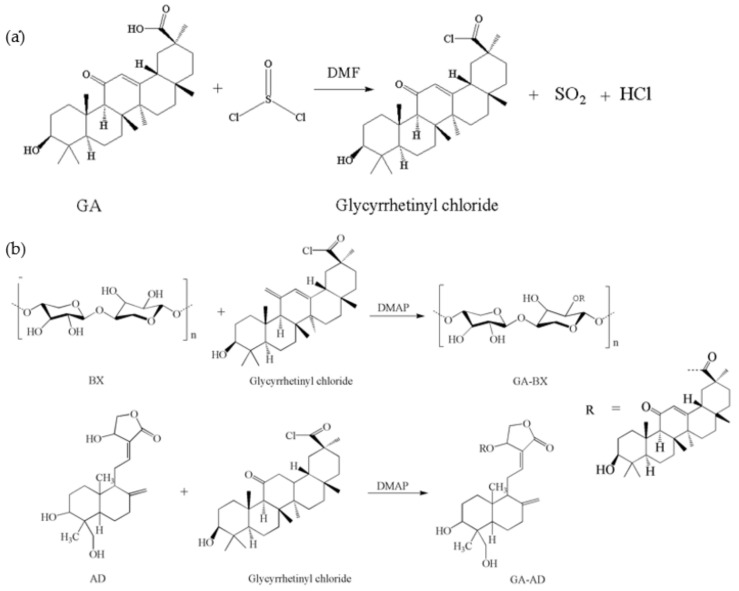

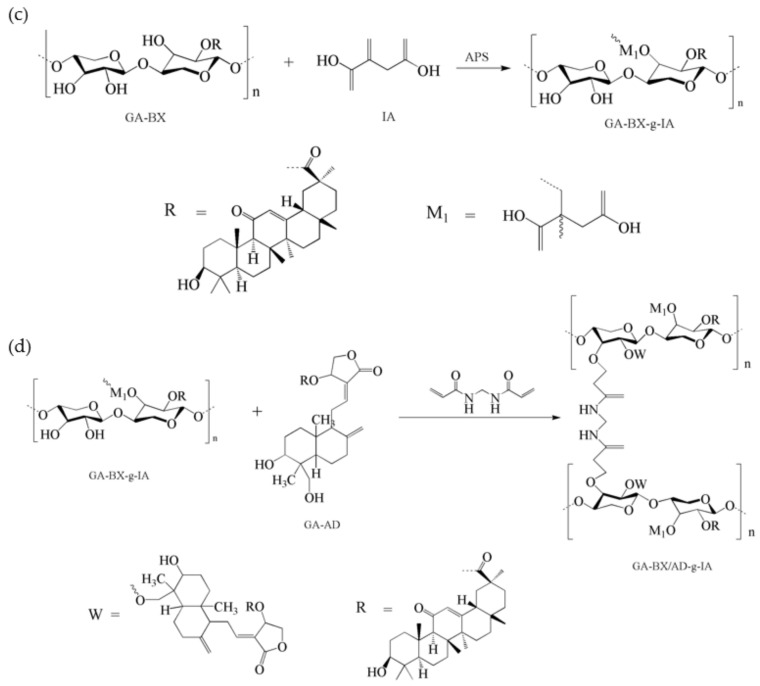
(**a**) The reaction mechanism of glycyrrhetinic acid chloride. (**b**) The reaction mechanism of GA-BX/AD. (**c**) The reaction mechanism of GA-BX-g-IA. (**d**) The reaction mechanism of GA-BX/AD-g-IA.

The glycyrrhetinic acid (3.0 g) was added to a four-mouth flask with dimedone 60 mL thionyl solution; then, the temperature was raised to 40 °C to dissolve the glycyrrhetinic acid, and then 0.46 mL DMSO and 0.30 mL DMF were added dropwise during constant stirring, with the mixture kept warm and stirred for 3 h to obtain a colorless, transparent, pungent-smelling liquid, which was glycyrrhetinic acid chloride (the reaction mechanism was shown in Figure 1a).

#### 2.2.2. Synthesis of Bagasse Xylan/Andrographolide Esterified Derivative

Figure 1b showed the reaction mechanism of the bagasse xylan/andrographolide esterified derivative. In a four-mouth flask containing glycyrrhetinic acid chloride, BX (1.34 g), AD (0.66 g), and catalyst DMAP (0.15 g) were added and the reaction was stirred continuously at 50 °C for 5 h and then cooled to room temperature (25 °C). The reaction product was precipitated with cyclohexane for 0.5 h. The reaction product was washed by filtration and the resulting filter cake was dried in a thermostat at 60 °C for 24 h to obtain the bagasse xylan/andrographolide esterified derivative (GA-BX/AD, 1.43 g).

#### 2.2.3. Synthesis of Bagasse Xylan/Andrographolide Grafted and Esterified Derivative

We configured the ammonium persulfate initiator solution and monomer solution separately. Firstly, 0.1–0.5 g of ammonium persulfate was dissolved completely with 5 mL of distilled water to obtain the initiator solution; then 0.5–3.0 g of itaconic acid was dissolved completely with 10 mL of distilled water to obtain the monomer solution.

GA-BX/AD (0.4 g) was added to a four-necked flask containing 50 mL of distilled water and stirred at 40 °C for 30 min. After that, MBAA (0.2 g) was added to it and the reaction was continued for 1–2 h. After the reaction was completed and cooled to room temperature (25 °C), the product was precipitated with acetone for 30 min and then filtered, washed three times, and dried at 60 °C for 24 h (the reaction mechanism is shown above in Figure 1c,d). 

The dried product was placed in a Soxhlet extractor, and 100 mL of acetone was added to it for 12 h to remove the monomeric auto polymer from the product, after which the product was dried at 60 °C for 24 h to obtain the bagasse xylan/andrographolide grafted and esterified derivative (GA-BX/AD-g-IA, 0.27 g).

#### 2.2.4. Synthesis of Bagasse Xylan/Andrographolide Grafted and Esterified Derivative Nanoparticles

At 80 °C, GA-BX/AD-g-IA (0.4 g) was added to a single flask and DMSO (40 mL) was added to dissolve it completely to make a 10 mg/mL solution of GA-BX/AD-g-IA. The solution was added to 400 mL of anhydrous ethanol and stirred continuously for 0.5 h. The bagasse xylan/andrographolide grafted and esterified derivative nanoparticles (GA-BX/AD-g-IA NPs, 0.23 g) were obtained by centrifugation, filtration, washing and drying to constant weight.

### 2.3. Determination of Degree of Substitution

Acid-base titration method [27] was the classical method for determining the degree of esterification substitution, and the specific experimental steps of the determination method were as follows:

1. Weigh 0.2 g of sample in a 50 mL conical flask;
2. Then add 10 mL of distilled water and 2 drops of phenolphthalein with a mass fraction of 5% to the conical flask and shake well;
3. Adjust the pH of the mixed solution to 7.0 with 0.5 mol/L of standard NaOH solution;
4. Add 2.0 mL of NaOH standard solution at a concentration of 0.5 mol/L, shaking well, and saponify with shaking at 25 °C for 4 h;
5. After saponification is complete, titrate the solution with a standard solution of HCl at a concentration of 0.5 mol/L to a pH of 7.0.

The degree of esterification substitution is calculated as follows:

(1) $$W_{c}=\frac{(V_{0}-V_{1})\times 10^{-3}\times C_{\mathrm{HCl}}\times M}{m}$$

(2) $$DS=\frac{168.5\times w_{c}}{M-\left(M-1\right)\times w_{c}}$$

where: *W_c_* is the mass fraction of carboxylic acid acyl groups in esterified derivatives, %; *V*_0_ is the volume of the HCl standard solution used for the blank experiment, mL; *V*_1_ is the volume of HCl standard solution used for titration of esterified derivatives, mL; *C*_HCl_ is the concentration of HCl standard solution, mol/L; *m* is the mass of the target product sample, g; *M* is the relative molecular mass of the carboxylic acyl group; 168.5 is the molar mass of BX/AD dehydration unit, g/mol; *DS* is the degree of esterification substitution.

### 2.4. Determination of Grafting Rate and Grafting Efficiency

The grafting rate and grafting efficiency of the product was calculated by mass method to evaluate the result of graft copolymerization [28]. Soxhlet extractor was used to extract for 24 h. Electronic scales were used to weigh the mass of the crude product before purification and the refined product after purification, and the grafting rate and grafting efficiency of the monomer were calculated through Formulas (3) and (4).

(3) $$G=\frac{W_{2}}{W_{0}}\times 100\%$$

(4) $$GE=\frac{W_{2}}{W_{1}}\times 100\%$$

where: *G* is grafting rate, %; *GE* is grafting efficiency, %; *W*_0_ is mass of BX/AD esterified derivative, g; *W*_1_ is mass of monomer, g; *W*_2_ is mass of grafted branched chain, g.

### 2.5. Characterization

The product was mixed with KBr in a certain ratio (1:30) to make thin slices and scanned with a Nicolet-iSL0 Fourier Transform Infrared Spectrometer in the range of 400–4000 cm^−1^ to obtain the infrared spectra. The surface morphology of the sample was observed using a Quanta 200 FEG Scanning Electron Microscope, the product was treated with gold plating and the morphology of the product was observed at different magnifications. The crystal structure of the sample was analyzed using a SmartLab 9 KW X-ray Diffractometer (Cu target, λ = 1.54056) with a scanning speed of 0.6565°/s and a scanning range of 5–90°. The thermal stability of the sample was tested using an SDT-Q600 Thermogravimetric Analyzer to determine the thermal degradation of the sample at 20–800 °C under the protection of nitrogen. The product was tested using a Bruker AVANCE III; 500 NMR Hydrogen Spectrometer at 500 MHz, 298.2 K.

### 2.6. Molecular Docking 

Molecular docking of GA-BX/AD-g-IA to relevant receptor proteins in the Protein Data Bank (PDB) was performed using the Lamarckian genetic algorithm using AutoDock docking software [29,30,31]. The structure of GA-BX/AD-g-IA was optimized by density flooding theory using Gaussian 09 software. The pure receptor protein structure was obtained by removing unwanted ligands, water, ions, and other small molecules from the receptor protein using Pymol software. The optimized product was hydrogenated and charged with the receptor proteins, respectively, and molecular docking simulations were performed to observe the best conformation of docking and theoretical analysis based on the binding free energy and binding constants.

### 2.7. Tumor Cell Proliferation Inhibitory Assay

The inhibition of GA-BX/AD-g-IA NPs on different cancer cells was evaluated by MTT assay [32]. The human liver cancer cells (BEL-7407), human breast cancer cells (MDA-MB-231), human gastric cancer cells (MGC80-3), and normal liver cells (LO_2_) used in this study were provided by Henan cancer hospital and the key laboratory of pharmaceutical chemistry and drug molecular engineering in Guangxi Normal University.

The above four types of cells were placed on 96-well plates and allowed to attach overnight. The four cells were treated with samples at concentrations of 1, 10, 20, 50, and 100 μg/mL, respectively, and the optical density was measured at 490 nm and 630 nm, with the former being the test group and the latter being the reference group. Among them, the blank experiment included only culture medium, MTT, and DMSO, and the sample experiment was material, cell culture medium, MTT, and DMSO. All experiments were repeated three times. The formulation of the inhibition ratio is shown in (5). The experimental results were analyzed using Microsoft Office Excel 2010, and the whole calculation was reported as mean ± standard value [33]. The formula for calculating the standard values is shown in (6).

Relative Cell Proliferation Ratio (RCR%):

(5) $$\;RCR\left(\%\right)=\frac{OD_{\left(sample,490nm-630nm\right)}-OD_{\left(blank,490nm-630nm\right)}}{OD_{\left(control,490nm-630nm\right)}-OD_{\left(blank,490nm-630nm\right)}}\times 100\%\;$$

Inhibition ratio = 1−RCR%

(6) $$SD=\sqrt{\frac{\sum_{i=1}^{n}{\left(x_{i}-x\right)}^{2}}{n-1}}$$

## 3. Results and Discussion

### 3.1. Analysis of the Results of the Single-Factor Experiment for DS

To improve the Degree of Esterification Substitution, the effects of reaction temperature, reaction time, the mass ratio of BX/AD to the catalyst, and mass ratio of BX/AD to glycyrrhetinic acid on the degree of product substitution were investigated and the synthesis conditions were optimized.

**Figure 2:**
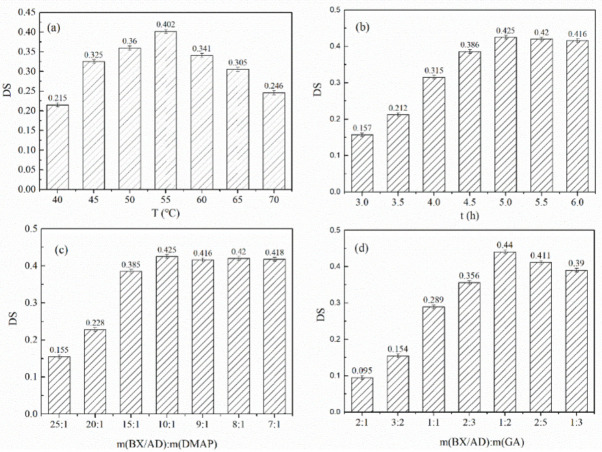
(**a**) Effect of reaction temperature on the *DS*. (**b**) Effect of reaction time on the *DS*. (**c**) Effect of catalyst dosage on the *DS*. (**d**) Effect of glycyrrhetinic acid dosage on the *DS*.

The effect of reaction temperature on the degree of esterification substitution is shown in Figure 2a. As the xylan crystalline region decomposes to create more active sites, increasing the temperature increases the reaction opportunity, but if the temperature was too high, the esterification reaction will proceed in the opposite direction, allowing the product to decompose, resulting in a lower degree of esterification substitution. Figure 2b showed the effect of reaction time on the degree of esterification substitution. As the number of hydroxyl active centers formed by the polymer saturates with increasing reaction time, the degree of esterification substitution also tended to level off with increasing reaction time. 

Figure 2c shows the effect of catalyst dosage on the degree of esterification substitution. The right amount of catalyst will promote the reaction, but due to the limited number of hydroxyl groups, a continuous increase in the amount of catalyst will not promote the formation of more esterification products. Therefore, the effect of catalyst dosage on the degree of esterification substitution tended to rise and then level off. Figure 2d showed the effect of the amount of glycyrrhetinic acid on the degree of esterification substitution. As the amount of glycyrrhetinic acid increases, the reaction proceeds positively, but when the active site became saturated, a further increase in the amount will lead to more esterification side reactions, resulting in a decrease in the degree of esterification substitution.

Therefore, a better synthesis process for the esterification stage was determined by single-factor experiments as follows: reaction temperature 55 °C, reaction time 5 h, m(BX/AD):m(DMAP) = 10:1, and m(BX/AD):m(GA) = 1:2.

### 3.2. Analysis of the Results of the Single-Factor Test for Grafting Rate (G) and Grafting Efficiency (GE)

The effects of reaction temperature, reaction time, initiator concentration, and amount of grafting monomer on grafting rate and grafting efficiency were investigated separately using the single-factor tests.

**Figure 3:**
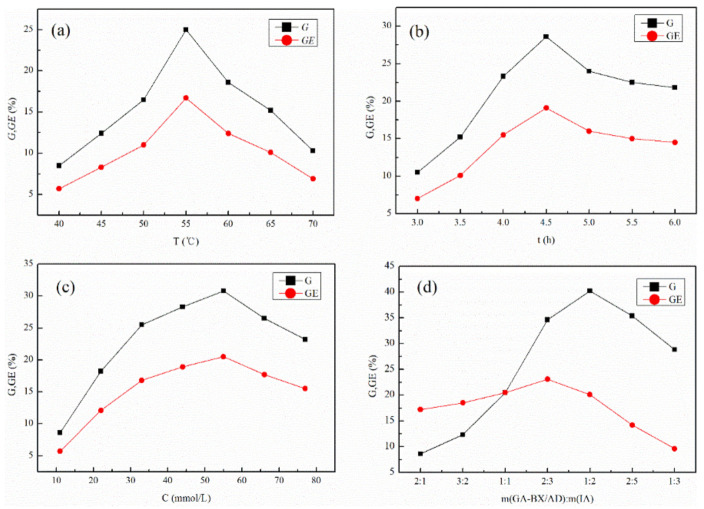
(**a**) Effect of reaction temperature on *G* and *GE*. (**b**) Effect of reaction time on *G* and *GE*. (**c**) Effect of initiator concentration on *G* and *GE*. (**d**) Effect of monomer quality on *G* and *GE*.

As shown in Figure 3a, the grafting rate and grafting efficiency of the products tended to increase and then decrease with increasing temperature. When the temperature was too high, it will lead to the generation of side reactions, resulting in the decomposition of the product and a decrease in the grafting rate and efficiency. As shown in Figure 3b, with the increase in reaction time, the grafting rate and grafting efficiency showed a trend of rapid increase and then slow decrease. As the reaction time increases, the concentration of the initiator decomposed into primary radicals gradually increases, the number of initiating xylan and monomer forming grafting sites increases, and the reaction process accelerates until the chain was terminated. 

Figure 3c showed that the grafting rate and grafting efficiency tend to increase and then decrease with increasing initiator concentration. The increasing initiator concentration will promote the reaction until chain termination, resulting in more self-polymerization of monomers, leading to an increase and then a decrease in grafting rate and grafting efficiency. As the number of monomers increases, the grafting rate and grafting efficiency increase and then decrease (as shown in Figure 3d). The increase in the amount of monomer will promote the reaction, but when the amount was too high, the monomer macromolecule will undergo self-polymerization, which was not conducive to the grafting reaction.

In summary, the results showed that the optimal synthesis process for the grafting reaction was: reaction temperature 55 °C, reaction time 4.5 h, initiator concentration 55 mmol/L, m(GA-BX/AD):m(IA) = 1:2.

### 3.3. Structure Analysis

#### 3.3.1. FTIR Analysis

**Figure 4:**
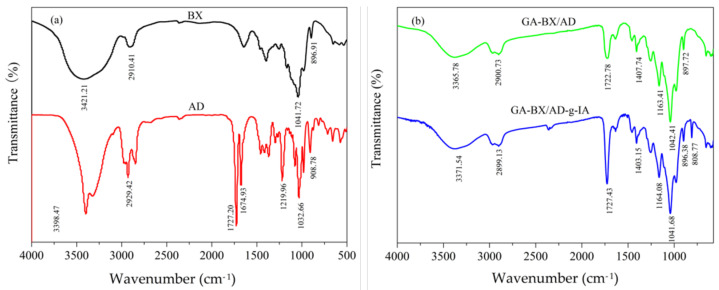
(**a**) FT-IR spectra of BX and AD. (**b**) FT-IR spectra of GA-BX/AD and GA- BX/AD-g-IA.

The functional groups of BX and AD and modified derivatives were characterized by FTIR to investigate the reaction between the raw materials and glycyrrhetinic acid, and itaconic acid. As shown in Figure 4a, for BX, the broad and strong absorption peak at 3421.21 cm^−1^ was assigned to the stretching vibration of —OH, and the peak at 2910.41 cm^−1^ was attributed to the stretching vibration of —CH_3_ and —CH_2_—, the C—O bond vibration peak at 1041.72 cm^−1^ and the stretching vibration peak of a β-D glycosidic bond at 896.91 cm^−1^. For AD, the peak at 3398.47 cm^−1^ was attributed to the stretching vibration of —OH, the absorption peaks at 1727.20 cm^−1^ and 1674.93 cm^−1^ were attributed to the stretching vibration of C=O, the absorption peaks around 1219.96 cm^−1^ and 1032.66 cm^−1^ were assigned to the ring lactones, and the absorption peak at 908.78 cm^−1^ was attributed to the stretching vibration of =CH_2_.

Figure 4b showed the FTIR spectra of GA-BX/AD and GA-BX/AD-g-IA. The characteristic peaks around 1722.78 cm^−1^ can be attributed to the carbonyl group of GA-BX/AD, the peak at 1407.74 cm^−1^ was attributed to the stretching vibration of —C(CH_3_)_2_, and the peak at 1163.41 cm^−1^ was the stretching vibration of the phenolic carbonyl group in glycyrrhetinic acid. Additionally, the introduction of IA made the peak more distinct. The peak at 1727.43 cm^−1^ was attributed to the stretching vibration of C=O, and 808.77 cm^−1^ was the stretching vibration peak of C—H in itaconic acid. Therefore, the characteristic groups of glycyrrhetinic acid and itaconic acid were introduced into the product. The spectra data of the BX, AD, GA-BX/AD, and GA-BX/AD-g-IA was shown in Table 1. 

#### 3.3.2. XRD Analysis

By comparing the proportions of peak diffraction and diffuse diffraction in the X-ray diffraction pattern, the crystalline properties of the GA-BX/AD-g-IA could be determined, and the change in the structure of the granules after esterification and grafting with glycyrrhetinic acid and itaconic acid could be understood.

**Figure 5:**
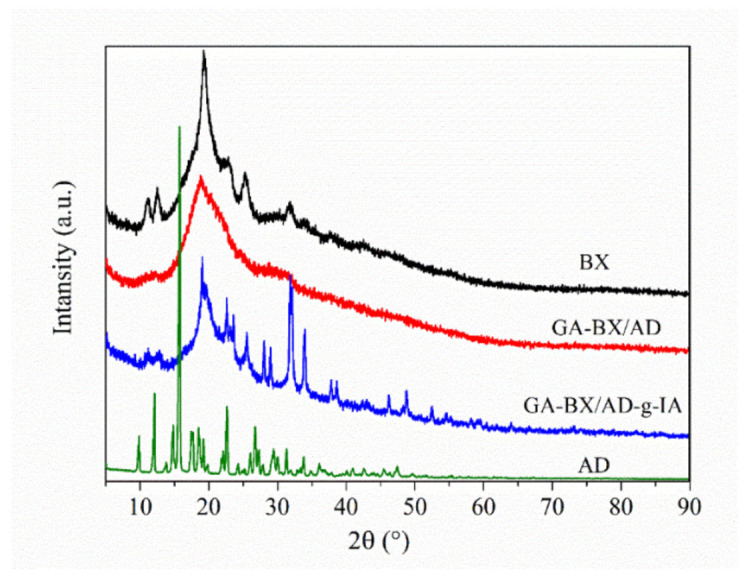
XRD of BX, AD, GA-BX/AD and GA-BX/AD-g-IA.

Figure 5 showed that the BX had obvious diffraction absorption peaks at 11°, 12.5°, 19.3°, 25.3°, and 31.7°. AD had Sharp and prominent characteristic absorption peaks at 12°, 15.7°, 22.6°, and 26.7°. This illustrated that the AD had high crystallinity and a complete crystallization region. After esterification, for the GA-BX/AD, the diffraction peak at 11°, 12.5°, 25.3°, and 31.7° disappeared, which indicated that esterification not only occurred in the amorphous region but also had an effect on the crystallization zone of the BX. This was mainly due to partial destruction of the crystallization zone of the BX and AD by degradation and esterification. By comparing the XRD patterns of GA-BX/AD-g-IA with BX and AD, it can be seen that GA-BX/AD-g-IA has a new diffraction peak at 23.5°, 28.9°, 31°, and 34°. The overall peak shape was high, narrow, and concentrated. In addition, the original diffraction peak of BX at 11° and 12.5° became weaker, and the peak deformation height at 31.7° became narrow. This indicated that the crystallization region of the modified product changed, the crystallinity increased, and the crystal region became larger.

#### 3.3.3. SEM Analysis 

**Figure 6:**
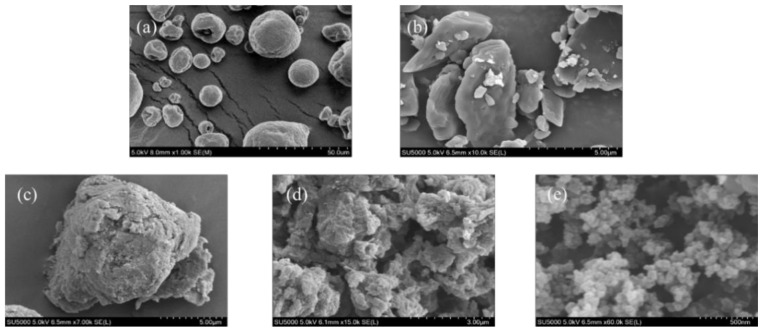
(**a**) The SEM of BX. (**b**) The SEM of AD. (**c**) The SEM of GA-BX/AD. (**d**) The SEM of GA-BX/AD-g-IA. (**e**) The SEM of GA-BX/AD-g-IA NPs.

To observe changes in surface morphology, native BX/AD and esterified BX/AD were scanned and characterized by a Scanning Electron Microscope (SEM), the results of which are shown in Figure 6a–e. Figure 6a shows that the BX granules were solid circles with the surface and edges both smooth. Figure 6b shows that the AD was flat in shape, with a smooth surface without damage and cracks.

Figure 6c shows the SEM of GA-BX/AD. Compared with the corn native BX and AD, the esterification reaction destroys the granule structure of the materials. There were morphology changes in the GA-BX/AD, the surface became rough and the granule surface was destroyed, which resulted from the increased length of xylan and glycyrrhetinic grafted to xylan granules. Moreover, in Figure 6d, the overall appearance of GA-BX/AD-g-IA is shown to be irregular with well-defined surface ravines and pronounced crevices. This illustrated that modification introduced glycyrrhetinic acid groups and itaconic acid monomers changed the surface topography of xylan and andrographolide. Figure 6e shows the surface morphology of GA-BX/AD-g-IA NPs as multiple spherical particles packed together with a particle size of about 100 nm.

The more regular morphology and smaller size of the product will be conducive to the dissolution and diffusion of the compound. At the same time, the morphological structure and size of the product will have an impact on the application field of the product. In particular, nanoparticles will promote the targeting delivery and absorption of drugs, thus broadening the research of the product in biomedicine and functional materials.

#### 3.3.4. DTG Analysis 

**Figure 7:**
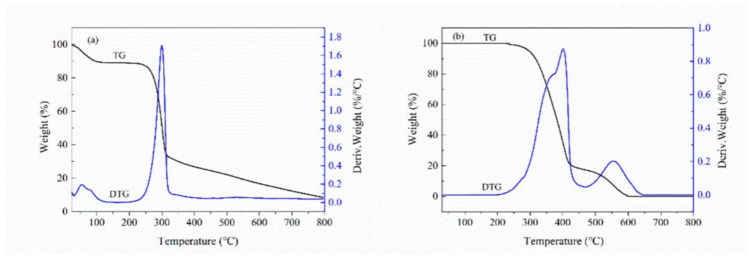
(**a**) DTG curves of BX. (**b**) DTG curves of AD.

The DTG curves of BX and AD are depicted in Figure 7. According to Figure 7a, three degradation stages were noticed for the BX. The first small weight loss occurred between 0 °C and 100 °C owing to the evaporation of absorbed water in BX. The second major weight loss arising in 220–320 °C was mostly caused by the breaking of glycosidic bond and hydroxyl bond in BX. The last weight loss was observed in the range of 320–800 °C, which was due to the residues on the BX backbone breaking. Moreover, the changes in the material due to the rising temperature for the AD sample were multistage (Figure 7b). Two mass losses were recorded on the TG curves of it: 250–450 °C and 450–600 °C. In the first stage, due to the breaking and decomposition of the five-membered lactone ring and triterpene double ring in AD, the mass decline rate was fast and the mass loss reaches 80%, and the second mass loss in the temperature range 450–600 °C was attributed to the fragmentation of —OH and C=C bonds.

**Figure 8:**
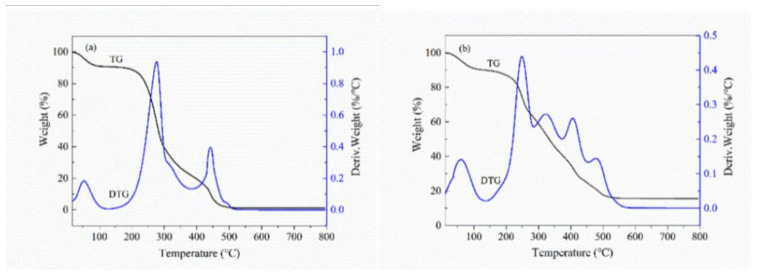
(**a**) DTG curves of GA-BX/AD. (**b**) DTG curves of GA-BX/AD-g-IA.

Figure 8a, b showed the DTG curves for GA-BX/AD and GA-BX/AD-g-IA. Their mass loss went through three stages. In Figure 8a, for GA-BX/AD, the first stage at 0–100 °C was attributed to the evaporation of water, residual acetone, and ethanol in the sample; the mass loss was 10%. The second stage within 200–380 °C was due to the broken of glycosidic bonds, and the last weight-loss stage at 380–500 °C was attributed to the rupture of the ester group of glycyrrhetinic acid and key bonds. In the case of GA-BX/AD-g-IA (Figure 8b), the weight loss during the first region (0–100 °C) was related to moisture removal; the next region (200–300 °C) could be explained by the main chains of BX and key bonds; and the large weight loss occurred within 300–500 °C as the temperature rose, indicating the thermal decomposition of ester bonds of the product.

The analysis of the TG and DTG curves showed that the mass loss of BX and AD decreases sharply with the increase of temperature in the interval of 200–600 °C, while the DTG curve of the product GA-BX/AD-g-IA shows more peaks, and the trend of mass loss was relatively slow in this interval, and there was still 18% remaining at 800 °C, indicating that the thermal stability of the product had been improved after modification.

#### 3.3.5. H NMR Analysis

**Figure 9:**
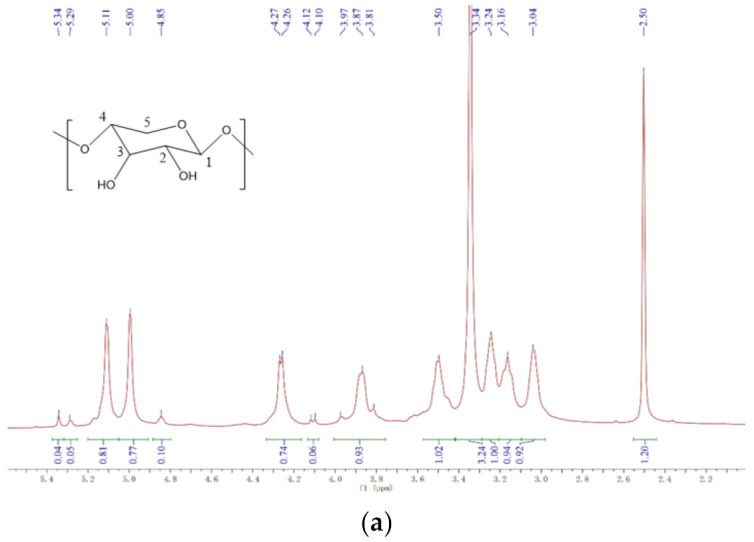

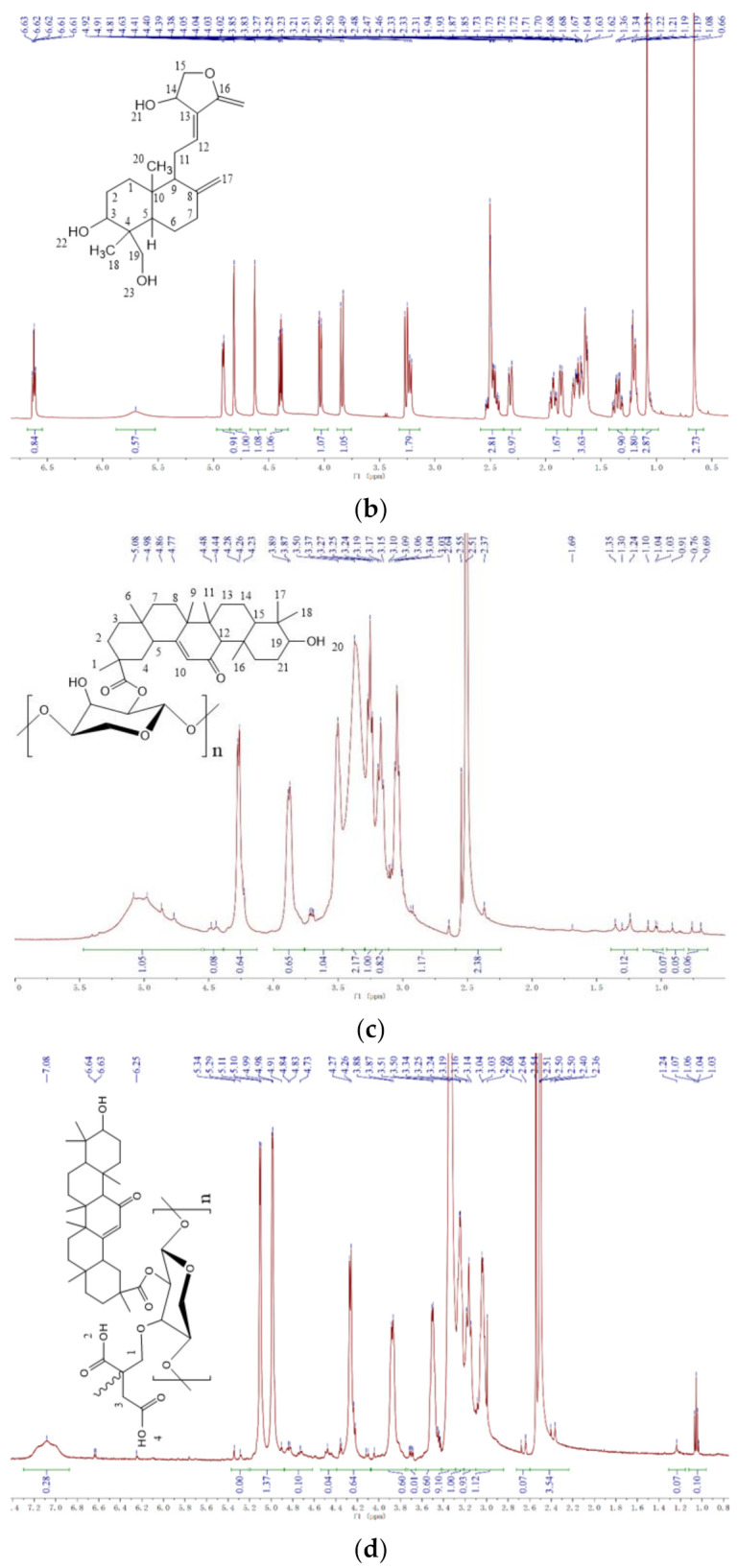
(**a**) The ^1^H NMR spectra of BX. (**b**) The ^1^H NMR spectra of AD. (**c**) The ^1^H NMR spectra of GA-BX/AD.(**d**) The ^1^H NMR spectra of GA-BX/AD-g-IA.

The ^1^H NMR spectra of BX is shown in Figure 9a,b. As shown in Figure 9a, the signal in the region from 3.04 to 5.34 ppm was the proton absorption peak of the BX xylose unit, the proton signal peak of H-1 at chemical shift 4.26 ppm, the absorption peak of H-2 at 3.16 ppm, the proton absorption peak of H-3 at 3.50 ppm, the signal peak at 3.81 ppm from H-4, and the chemical shift absorption peaks at 3.24 ppm and 3.87 ppm were produced by H-5. The distribution of the chemical shift to which the individual protons of AD belong in Figure 9b was as follows: the proton absorption peak of H-12 at 6.55–6.67 ppm, which was due to the presence of olefinic protons in α, β-unsaturated lactones. The absorption peak at 4.86–4.96 ppm was attributed to H-14, the exocyclic methylene proton H-17 resonated as a single linear state at 4.74-4.86 ppm and 4.57-4.67 ppm. and the absorption peaks of H-15 were at 4.32–4.45 ppm and 3.97–4.09 ppm, H-19 at 3.76–3.89 ppm. The absorption peaks of H-1, H-2, and H-6 were present at 2.24–2.37 ppm.

From Figure 9c, the absorption peaks of GA-BX/AD at 3.15 ppm belonged to the proton peaks of H-19 in glycyrrhetinic acid, at 1.35 ppm and 1.10 ppm to the proton peaks of H-7 in glycyrrhetinic acid, and at 1.30 ppm and 1.04 ppm to the proton absorption peaks of H-9, H-11 in glycyrrhetinic acid of —CH_3_, respectively. In Figure 9d, in addition to the proton peaks of GA-BX/AD, the proton absorption peaks of H-1 occurred at 6.90–7.30 ppm. From these proton absorption peaks, and combined with infrared spectroscopic analysis, it was known that glycyrrhetinic acid and itaconic acid groups were successfully introduced into the products.

#### 3.3.6. Molecular Docking Analysis

**Figure 10:**
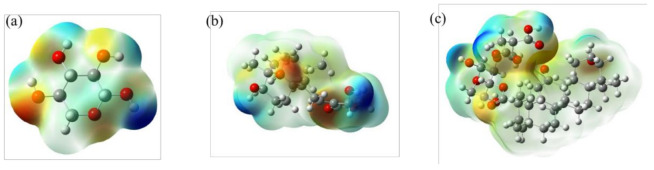
Electrostatic potential distribution of BX (**a**), AD (**b**) and GA-BX/AD-g-IA (**c**).

To investigate the anticancer activity of the product, we docked GA-BX/AD-g-IA with some of the receptor proteins (6CFN, 2W4Q, 3EAE, 6IIQ) using AutoDock, Pymol, and Gaussian 09 software. Usually, the binding free energy and the binding constant of docking are important parameters to evaluate the effect of molecular docking. Among them, the relevant docking parameters are shown in Table 2. Moreover, the Electrostatic potentials distribution of BX (a), AD (b), and GA-BX/AD-g-IA(c) were shown in Figure 10 (the negative potentials were shown in red with proton attraction and the positive potentials were shown in blue with proton repulsion). The results showed that GA-BX/AD-g-IA had a certain affinity with all four receptor proteins, among which the docking effect with the 6CFN protein was more significant. Therefore, the product had a theoretical anti-cancer effect, which will be subsequently confirmed by MTT assay.

**Figure 11:**
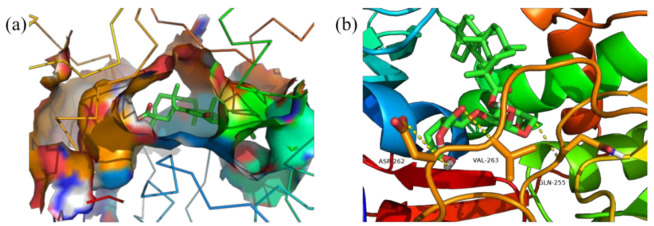
Docking conformation diagram (**a**) and hydrogen bonding diagram (**b**) of GA-BX/AD-g-IA with 2W4Q.

**Figure 12:**
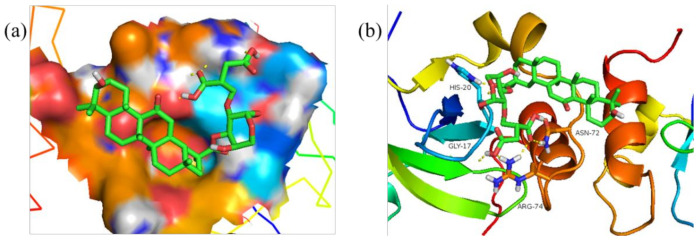
Docking conformation diagram (**a**) and hydrogen bonding diagram (**b**) of GA-BX/AD-g-IA with 3EAE.

**Figure 13:**
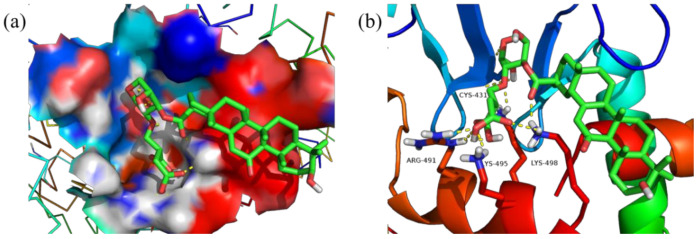
Docking conformation diagram (**a**) and hydrogen bonding diagram (**b**) of GA-BX/AD-g-IA with 6CFN.

**Figure 14:**
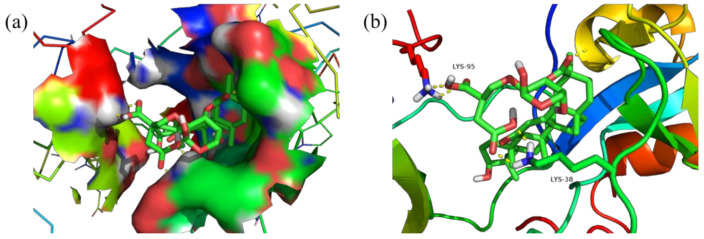
Docking conformation diagram (**a**) and hydrogen bonding diagram (**b**) of GA-BX/AD-g-IA with 6IIQ.

Meanwhile, Figure 11, Figure 12, Figure 13 and Figure 14 show the best docking conformation of the product with the four receptor proteins, from which it can be found that GA-BX/AD-g-IA and some amino acid residues of 2W4Q, 3EAE, 6CFN, and 6IIQ undergo hydrogen bonding, in the binding pocket of the proteins. Among them, GA-BX/AD-g-IA produced hydrogen bonds with residues ASP-262, VAL-263, and GLN-255 of 2W4Q; with residues HIS-20, GLY-17, ASN-72, and ARG-74 of 3EAE; and with residues CYS-431, ARG-49, LYS-495, and LYS-498 of 6CFN hydrogen bonding. Hydrogen bonds were produced with residues LYS-38 and LYS-95 of 6IIQ, which indicated that GA-BX/AD-g-IA had high binding stability and good docking with the four receptor proteins.

#### 3.3.7. Inhibition Analysis of Tumor Cell 

The inhibition ratio of human liver cancer cells (BEL-7407), human breast cancer cells (MDA-MB-231), human gastric cancer cells (MGC80-3), and human normal liver cells (LO_2_) at different sample concentrations were determined by the MTT assay. The results of the experiments are shown in Table 3. The results showed that the inhibition ratio of BX/AD on liver cancer cells was 2.37 ± 0.73% when the sample mass concentration was 100 μg/mL, while the inhibition rate of GA-BX/AD-g-IA NPs was up to 38.41 ± 5.32%, which was about 16 times higher than that of BX/AD. Meanwhile, the inhibition ratio of the product on the three cancer cells was gradually enhanced along with the increment of mass concentration, and it did not affect human normal liver cells. This indicates that the introduction of glycyrrhetinic acid and itaconic acid will effectively improve the anticancer activity of BX and AD complex. Meanwhile, the bagasse xylan/andrographolide grafted and esterified derivative nanoparticles can improve their solubility and diffusivity for better application in targeted therapy.

In addition, we compared the inhibitory effects of Niclosamide and Xyl-LA/Nic NPs on human colorectal adenocarcinoma cells (HCT-15) using the MTT assay. Among them, the IC_50_ of Xyl-LA/Nic NPs on HCT-15 was 4.2 ± 1.6, the IC_50_ of clonidine on HCT-15 was 7.6 ± 2.4, and the IC_50_ value of Xyl-LA/Nic NPs was about 1.8 times lower than Niclosamide. It was shown that Xyl-LA/Nic NPs have some anticancer activity [34,35]. Meanwhile, the product GA-BX/AD-g-IA NPs obtained in this study had better inhibitory effects on human liver cancer cells, human breast cancer cells, and human gastric cancer cells especially, and its inhibition ratio of liver cancer cells (BEL-7407) was about 38 times higher than that of xylan, showing excellent anticancer activity.

## 4. Conclusions 

In summary, a novel bagasse xylan/andrographolide esterified graft derivative with anticancer activity was synthesized in this paper. The better synthesis process was determined by single-factor experiments. FTIR, XRD, DTG, SEM, and ^1^H NMR were used to characterize the structure, stability, and morphology of the product. The docking effect and anticancer activity of the derivative with the receptor protein were measured and evaluated by molecular docking technique and MTT assay.

The results showed that the esterification degree of substitution was 0.43 and the grafting rate was 42% under a better synthesis process. Compared with BX and AD, the apparent morphology of GA-BX/AD-g-IA NPs changed significantly, with smaller particle size and particle buildup. With the increase in temperature, they showed a slowly decreasing trend of mass loss and improved thermal stability by comparison with to BX and AD. Additionally, the product showed a good docking effect with the four receptor proteins. Combined with FTIR and anti-cancer activity data analysis, the product contained the active groups of glycyrrhetinic acid and itaconic acid. Moreover, at the same mass concentration (10 μg/mL), the inhibition ratio of BX against hepatocellular carcinoma cells (BEL-7407) was 0.47 ± 0.29%, and the inhibition ratio of BX/AD was 0.93 ± 0.34%, while the inhibition ratio of GA-BX/AD-g-IA NPs was 26.74 ± 4.29%, which was about 28 times higher than that of BX/AD, with good anti-cancer effect. Meanwhile, compared with BX, GA-BX/AD-g-IA NPs also showed significant inhibitory effects on gastric cancer cells and breast cancer cells and did not affect human normal liver cells. This indicates that the physical properties and anticancer activity of the product were enhanced after compounding, grafting, esterification, and cross-linking. These improvements in physicochemical properties will broaden the application of Xylan/Andrographolide grafted and esterified derivative nanoparticles (GA-BX/AD-g-IA NPs) in biopharmaceutical applications.

Based on these results, GA-BX/AD-g-IA has good anticancer activity, which gives it potential for application in the field of biomass anticancer. Meanwhile, this paper provides a new idea for the preparation and application of functional materials.

## Figures and Tables

**Table 1:** FT-IR spectra data of BX, AD, GA-BX/AD, and GA-BX/AD-g-IA.

Assignment	Frequency (cm^−1^)			
	BX	AD	GA-BX/AD	GA-BX/AD-g-IA
Hydroxy bond	3421.21	3398.47	3365.78	3371.54
Methyl or methylene group	2910.41	2929.42	2900.73	2899.13
Ester carbonyl	/	1727.20	1722.78	1727.43
Alkyl group	1397.85	/	1407.74	1403.15
C—O stretching of phenolic	1041.72	1219.96	1163.41	1164.08
C—H stretching vibration peak	896.61	908.78	897.72	895.23
C—H bond of IA	/	/	/	808.77

**Table 2:** The dock evaluation of receptor protein with GA-BX/AD-g-IA.

PBD Code	Estimated Free Energy of Binding (kcal/mol)	Ki (μM)	Final Intermolecular Energy (kcal/mol)	Final Total Internal Energy (kcal/mol)
2W4Q	−13.65	98.08 × 10^−6^	−17.83	−2.82
3EAE	−8.94	280.61 × 10^−3^	−13.11	−2.49
6CFN	−14.68	17.46 × 10^−6^	−18.85	−2.75
6IIQ	−10.35	25.82 × 10^−3^	−14.53	−3.12

**Table 3:** The inhibition ratio of BX, BX/AD and GA-BX/AD-g-IA on different cancer cells and normal cells.

Sample	Mass Concentration/(μg/mL)	Inhibition Ratio/%			
		LO_2_	BEL-7407	MDA-MB-231	MGC80-3
BX	100	1.68 ± 0.50	1.07 ± 0.71	3.16 ± 0.94	2.02 ± 0.57
	50	1.15 ± 0.77	1.18 ± 0.34	2.35 ± 0.72	0.24 ± 0.08
	20	−0.81 ± 0.79	0.35 ± 0.26	1.62 ± 0.47	−0.15 ± 0.13
	10	−3.34 ± 0.31	0.47 ± 0.29	0.98 ± 0.33	−2.99 ± 1.11
	1	−6.98 ± 0.29	−0.45 ± 0.31	0.17 ± 0.12	−3.27 ± 1.61
BX/AD	100	1.26 ± 0.79	2.37 ± 0.73	5.62 ± 1.43	4.28 ± 1.26
	50	0.83 ± 0.61	2.42 ± 0.81	3.85 ± 0.61	2.35 ± 0.71
	20	−1.75 ± 1.02	1.28 ± 0.65	2.08 ± 0.29	1.73 ± 0.49
	10	−5.21 ± 2.23	0.93 ± 0.34	1.67 ± 0.50	0.68 ± 0.35
	1	−7.49 ± 0.38	−0.74 ± 0.69	0.83 ± 0.26	−1.23 ± 0.84
GA-BX/AD-g-IA	100	2.84 ± 0.57	38.41 ± 5.32	26.92 ± 4.25	32.69 ± 4.87
	50	2.03 ± 0.75	35.76 ± 5.11	23.47 ± 3.86	29.46 ± 3.91
	20	1.14 ± 0.64	30.49 ± 4.02	20.15 ± 2.47	26.37 ± 4.18
	10	0.32 ± 0.20	26.74 ± 4.29	16.44 ± 2.01	22.15 ± 3.36
	1	−1.89 ± 0.68	20.16 ± 1.95	11.38 ± 1.64	17.25 ± 1.83

## Data Availability

Not applicable.
